# Renal Paraganglioma: Report of a Case Managed by Robotic Assisted Laparoscopic Partial Nephrectomy and Review of the Literature

**DOI:** 10.1155/2014/527592

**Published:** 2014-05-05

**Authors:** Burak Bahar, Stefan E. Pambuccian, Gopal N. Gupta, Güliz A. Barkan

**Affiliations:** ^1^Department of Pathology, Loyola University Medical Center, South First Avenue, Maywood, IL 60153, USA; ^2^Departments of Urology, Surgery and Radiology, Loyola University Medical Center, South First Avenue, Maywood, IL 60153, USA

## Abstract

We describe the pathological and clinical presentation of a rare case of renal paraganglioma occurring as an incidental left renal mass in a 58-year-old woman. The patient underwent robotic assisted laparoscopic partial nephrectomy, which is the first one in the literature.

## 1. Introduction

Paragangliomas are tumors that arise from neural crest tissue and can be found throughout the body. The World Health Organization advocates using the term “paraganglioma” for extra-adrenal tumors, while the term “pheochromocytoma” is restricted to tumors with similar gross, microscopic, clinical, and molecular features originating from the adrenal glands [1]. About 10% of paragangliomas occur in extrarenal location; about half of these arise from the organs of Zuckerkandl and most of the remainder from the retroperitoneum. Rare sites of occurrence of paragangliomas are the bladder, urethra, prostate and seminal vesicles, and the kidneys [2]. Renal paragangliomas are not only rarely encountered, but also difficult to distinguish clinically and pathologically from renal cell carcinoma. Both paragangliomas and pheochromocytomas are characterized grossly by the brown appearance of the cut surface and microscopically by the presence of small nests of uniform polygonal chromaffin cells (Zellballen), rimmed by variable amounts of spindled “sustentacular” cells and surrounded by capillaries. Since there is no consensus on staging, both tumors are divided into three categories: benign (localized), regional, and metastatic. Paragangliomas generally follow a benign clinical course with the famous quoted incidence of malignancy as 10%. When possible, total excision of paragangliomas is the first treatment modality and is curative in benign cases. We herein report the clinical, imaging, and pathologic features of a case of intrarenal paraganglioma, which is, to the best of our knowledge, the tenth reported renal paraganglioma and the first to be managed by robotic assisted laparoscopic partial nephrectomy.

## 2. Case Report

**Figure 1:**
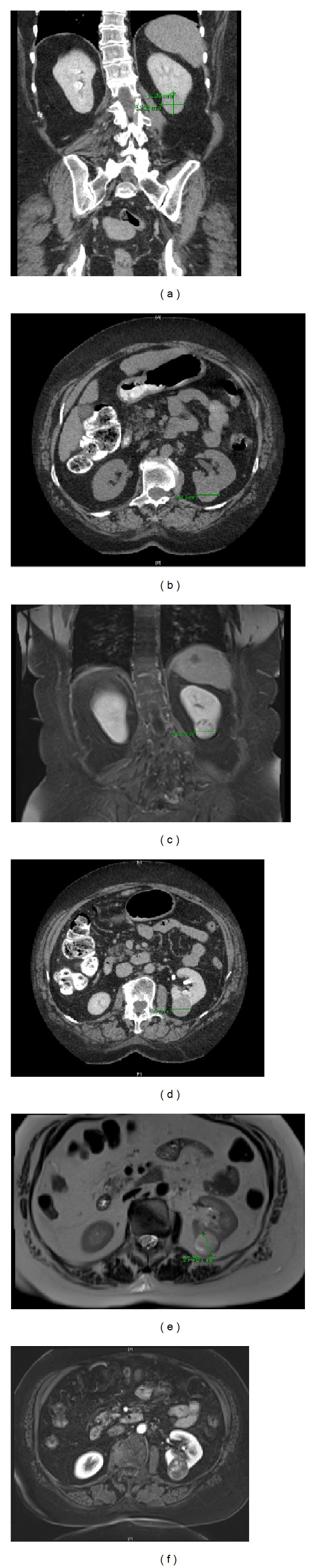
(a)–(c) CT revealed a solid tumor arising from the posterior cortex of the kidney near the inferior pole. The tumor measured 3.5 cm in greatest diameter. (d)–(f) MRI revealed an exophytic mass arising from the posterior cortex of the kidney near the inferior pole. The mass demonstrated pouch and its internal enhancement, heterogeneous high T2 signal, and restricted diffusion.

A 58-year-old woman with a history of autoimmune hepatitis and primary biliary cirrhosis presented for further evaluation and management of a left renal mass that was incidentally discovered during routine abdominal ultrasound. Abdominal CT scan and MRI studies showed an exophytic mass arising from the left posterior lower pole renal cortex, measuring 3.5 × 3.0 cm. The tumor was abutting the renal collecting system without causing hydronephrosis (Figures 1(a) and 1(b)). The left renal vein and inferior vena cava were patent, and there was no significant retroperitoneal or mesenteric adenopathy. The adrenal glands were normal in appearance. On further inquiry, the patient admitted occasional right flank pain but denied hematuria. Physical examination of the patient was within normal limits; no abdominal mass could be palpated. No history of hypertension or headaches was elicited and the blood pressure was normal. A provisional diagnosis of renal cell carcinoma was made and the patient underwent robotic assisted laparoscopic retroperitoneal left partial nephrectomy. The tumor was enucleated in its entirety.

The partial nephrectomy material was examined grossly and microscopically. The excision specimen was fixed in 10% neutral buffered formalin and embedded in paraffin. 5 *μ*m sections were made and stained with hematoxylin and eosin. Immunohistochemical (IHC) stains were performed on the tissue section. IHC analyses were performed on the Benchmark XT automated immunostaining module (Ventana Medical Systems, Tucson, AZ). Positive control tissue was placed on each slide, and the following antibodies were used: synaptophysin (27G12, Leica, Buffalo Grove, IL), chromogranin (5H7, Leica), CD56 (CD564, Leica), S-100 (Polyclonal, Leica), CK7 (RN7, Leica), pan-cytokeratin (AE1/AE3, Leica), vimentin (SRL33, Leica), smooth muscle actin (1A4, Ventana, Tucson, AZ), and HMB-45 (HMB-45, Leica).

**Figure 2:**
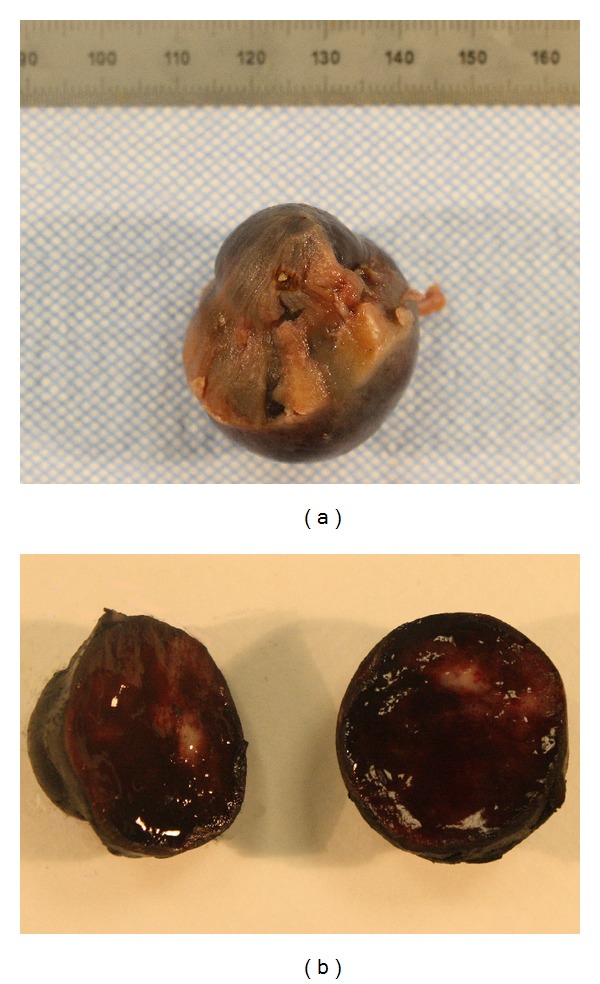
(a) At external inspection, tumor is blue-black and capsular surface is thickened. (b) In cross section, tumor is dark red and hemorrhagic with an irregular area of white-tan parenchyma, 2.4 cm in greatest dimension.

**Figure 3:**
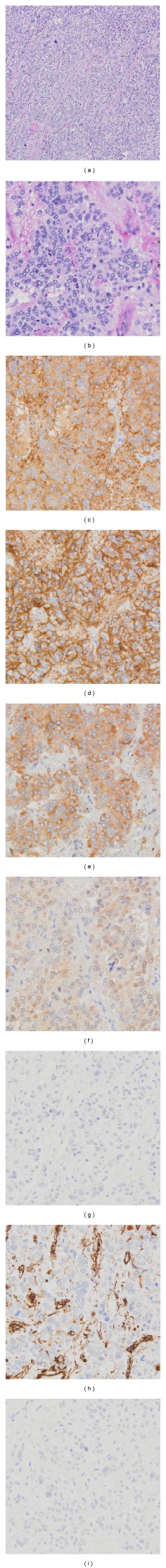
Intrarenal paraganglioma. Low magnification (a) and high magnification (b) of tumor cells (hematoxylin and eosin). Tumor cells forming a “Zellballen pattern” with interspersed thin-walled vascular structures. IHC showed tumor cells are positive for synaptophysin (c), CD 56 (d), and chromogranin (e). Sustentacular cells were positive for S-100, although tumor cells were negative (f). The tumor cells were negatively stained for HMB45 (g), vimentin (h), and CK7 (i).

Macroscopic examination of the partial nephrectomy specimen revealed a dark red, hemorrhagic, moderately firm, well-circumscribed mass, measured 3.4 × 3.2 × 2.7 cm. The mass was contained within the renal capsule, which it abutted (Figure 2). The partial nephrectomy margins were negative. Microscopically, the tumor disclosed the characteristic, well defined nests of cuboidal cells forming “Zellballen” that were peripherally encircled by spindle cells (sustentacular cells). The cell nests were separated by highly vascularized fibrous septa (Figures 3(a) and 3(b)). No nuclear pleomorphism, necrosis, or mitoses were identified. IHC analysis showed that tumor cells were strongly and diffusely positive for synaptophysin, chromogranin, and CD56 and were negative for pan-cytokeratin, CK7, vimentin, smooth muscle actin, and HMB-45. S-100 highlighted the sustentacular cells (Figures 3(c) to 3(i)). A diagnosis of intrarenal paraganglioma was made based on these morphologic and immunohistochemical findings.

## 3. Discussion

In the case presented herein, the clinical and imaging findings suggested renal cell carcinoma—the most common type of solid kidney tumor in adults who have a solitary lesion with enhancement on CT and hyperintensity with heterogeneity on T2-weighted MRI [3]—but the histologic and IHC findings were characteristic of an intrarenal paraganglioma.

The tumor was confined to the kidney without any regional lymphatic involvement or distant metastasis. The tumor showed no significant mitotic activity and no necrosis and lacked sheetlike growth and nuclear pleomorphism, with all histologic features favoring a benign paraganglioma. In addition, the tumor showed a well-represented sustentacular cell component, a feature which has been shown to be associated with a benign outcome [4]. Application of the PASS scoring system that was proposed to separate benign from malignant pheochromocytomas showed a score of <4, which also suggested a benign tumor [5]. Nonetheless, histology is an imperfect tool to predict the behavior of paragangliomas, as there are no absolute criteria of malignancy apart from the presence of invasion and metastasis, and no paraganglioma or pheochromocytoma can be considered unequivocally benign [2].

A thorough literature review found only twelve additional cases of intrarenal paraganglioma reported up to date, occurring in 6 men and 7 women with a mean age of 42.6 years. In the older literature, some of these cases were reported as “renal pheochromocytoma” in accordance with the nomenclature used at the time. The exact mechanism by which paragangliomas occur in the kidney is unknown; one theory suggests an origin in ectopic adrenal tissue or adrenal rests located in the kidney [6]. Ectopic adrenal tissue is caused by multiple adrenal primordia or from fragmentation of the adrenal primordia during embryogenesis; the more commonly found ectopic adrenal rests that migrate with the developing gonads consist only of cortical tissue, while the ectopic adrenal rests situated close to the original position of the adrenal gland may also contain medulla. Another possible explanation suggested for the occurrence of intrarenal paragangliomas is renal-adrenal fusion, in which the heterotopic adrenal gland is entirely contained within the renal capsule. However, such cases show only adrenal cortical tissue and lack adrenal medulla [7]. In most of the reported cases of renal paraganglioma, the ipsilateral adrenal gland was identified within the nephrectomy specimen and was morphologically unremarkable.

Renal paragangliomas have been reported to arise in the upper pole or hilum or, as in the case presented herein, from the lower pole of the kidney. The location of the tumors within the kidney appears to be an important determinant of the patients' clinical presentation. Tumors located in the renal pelvis/hilum have been reported to cause hypertension renal artery stenosis [8]. The reported sizes of the tumors varied from 2.5 to 18 cm; larger tumors were cystic. The size of these tumors is similar to the size of renal cell carcinomas. The relatively large size of the tumors may be ascribed to their lack of symptoms, since even small paragangliomas (and pheochromocytomas) can be detected early if they cause symptoms like palpitations, sweating, and headaches due to hormone hypersecretion.

Like in our case, many of the reported cases of renal paragangliomas were not associated with hypertension or other symptoms of catecholamine hypersecretion and were labeled as nonsecretory. However, determination of urine catecholamines was not performed in most cases, since the tumors were clinically diagnosed as renal cell carcinomas. None of the reported cases was diagnosed as or suspected to be paraganglioma or pheochromocytoma preoperatively. The presence of hypertension, especially when encountered in a younger patient or associated with paroxysmal headaches and palpitations, may raise the suspicion of paraganglioma or pheochromocytoma; however, in the presence of a renal tumor on imaging studies, these signs and symptoms may be attributed to renal cell carcinoma, which may also be associated with hypertension. Preoperative core needle biopsy or fine needle aspiration may provide a preoperative diagnosis; however, biopsies are generally contraindicated in cases suspected to represent paraganglioma or pheochromocytoma as the procedure may lead to potentially fatal complications.

Metastases were present in 6 cases [8–12] and occurred after a prolonged interval [9, 10, 12]. Like in our case, most reported cases of renal paraganglioma showed characteristic histologic features and, when performed, a typical immunostaining pattern. However, some reported cases showed unusual histologic or IHC features, such as pigmentation [6], predominance of spindle cells [11], or absence of sustentacular cells [6].

Like in the patient presented herein, based on the imaging studies and clinical findings, the patients' preoperative diagnosis or suspicion was renal cell carcinoma in all reported cases. Renal cell carcinoma must be considered in the differential diagnosis of every renal mass lesion, even in incidentally detected solid renal masses, since such incidentally discovered renal masses account for about 50% of all currently diagnosed RCCs [13]. Current imaging techniques cannot reliably determine malignancy in a renal mass [12], although the likelihood of malignancy is increased by certain imaging characteristics such as size, shape, contours, and contrast enhancement. Therefore, all incidentally discovered solid renal masses have to be considered malignant unless proven otherwise and are frequently managed by nephron-sparing surgery. A preoperative biopsy may be used in some cases to guide management, especially when the imaging features are atypical, but the rate of nondiagnostic biopsies is high in small masses [14].

Because of the relative frequency of renal cell carcinoma among resected renal masses, its numerous histologic variants, and variable histologic appearance, renal cell carcinoma is also the first diagnosis considered by the pathologist. Histologically, renal cell carcinomas show some similarities with paragangliomas: both are composed of polygonal to spindle cells growing in nests and surrounded by a rich capillary network; both can be composed of cells showing cytoplasmic clearing or oncocytic cytoplasm, occasional cytoplasmic hyaline globules, and relatively monotonous bland nuclei. However, paragangliomas tend to have less abundant cytoplasm, show salt-and-pepper chromatin, and usually have less nuclear pleomorphism and less prominent nucleoli than renal cell carcinomas. Another feature that may allow the pathologist to suspect a paraganglioma is the presence of basophilic or amphophilic rather than eosinophilic granular or clear cytoplasm. Once the diagnosis of paraganglioma is suspected, immunostains can be performed to aid in this differential diagnosis. In contrast to renal cell carcinomas, most extra-adrenal paragangliomas are negative or only weakly or focally positive for keratins and are diffusely positive for neuroendocrine markers (synaptophysin, chromogranin, and CD56) [15]. A novel marker in the diagnosis of paragangliomas is GATA3 that is expressed in about 80% of all cases regardless of site [12]. The identification of S-100-positive sustentacular cells is also of diagnostic value in differentiating paragangliomas from renal cell carcinomas and can, in addition, help differentiate renal paragangliomas from low grade neuroendocrine tumors that may also be rarely encountered in the kidney [16]. The difficulty in differentiating paragangliomas from renal cell carcinomas is illustrated by the case reported by Takahashi et al., which was retrospectively diagnosed as paraganglioma as a result of an unrelated cDNA microarray research study of cases originally diagnosed as renal cell carcinoma [17].

The differential diagnosis of paragangliomas with unusual histologic features, such as those composed predominantly of spindle cells or showing pigmentation, also includes sarcomatoid renal cell carcinoma, leiomyosarcoma, peripheral nerve sheath tumor, neuroepithelial tumor, mesoblastic nephroma, myopericytoma, angiomyolipoma, and melanomas. The differentiation of primary renal paragangliomas from the more common renal metastases from pheochromocytomas and paragangliomas is based on imaging studies [10].

To our knowledge, this is the first case of renal paraganglioma to be treated with robotic assisted surgery. No intraoperative hypertension or other anesthetic complications were noted. The successful use of robotic partial nephrectomy in this case demonstrates that this method is as useful in the management of renal paragangliomas as it is in the management of adrenal pheochromocytomas [18]. The patient's postoperative course was free of complications and imaging studies did not show any recurrence or metastasis during a follow-up period of six months [18, 19].

In summary, we report a very rare location of paraganglioma and the first one to be managed by robotic assisted partial nephrectomy. The lack of operative complications and uneventful postoperative course support the use of robotic assisted surgery for suitable candidates with such unusual tumors.
